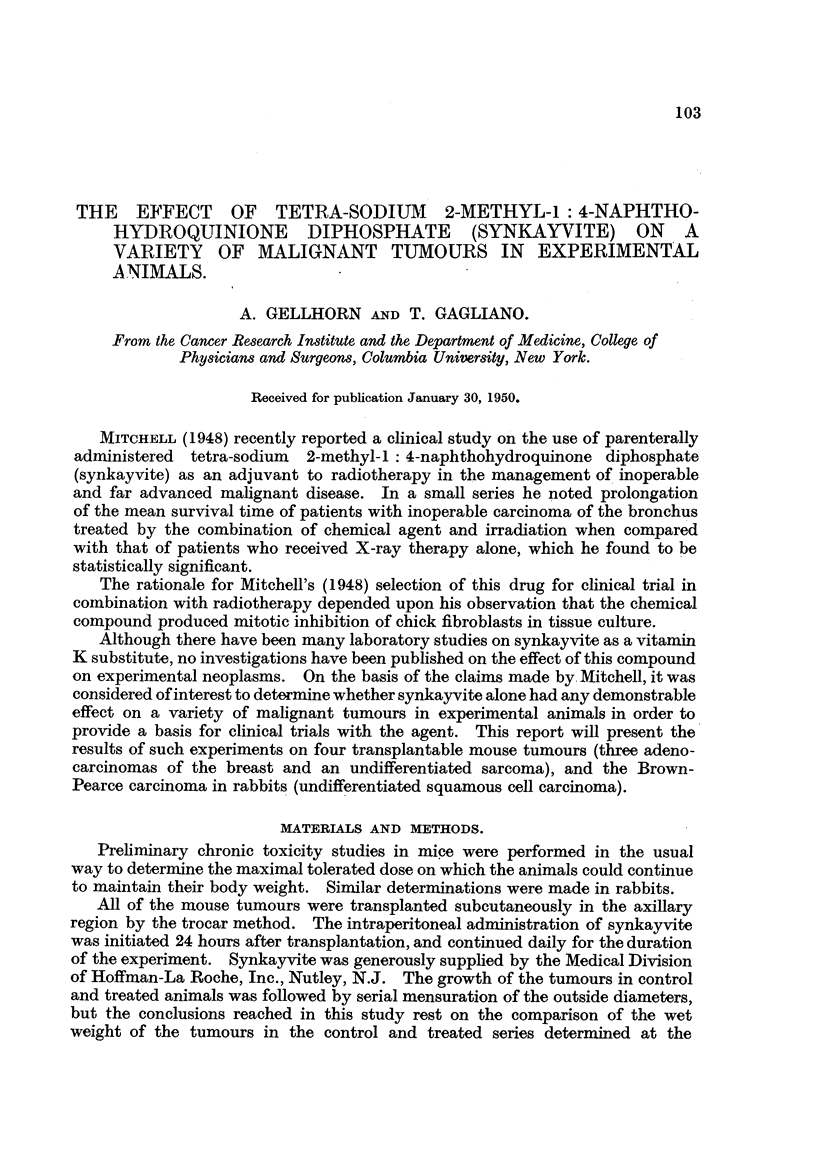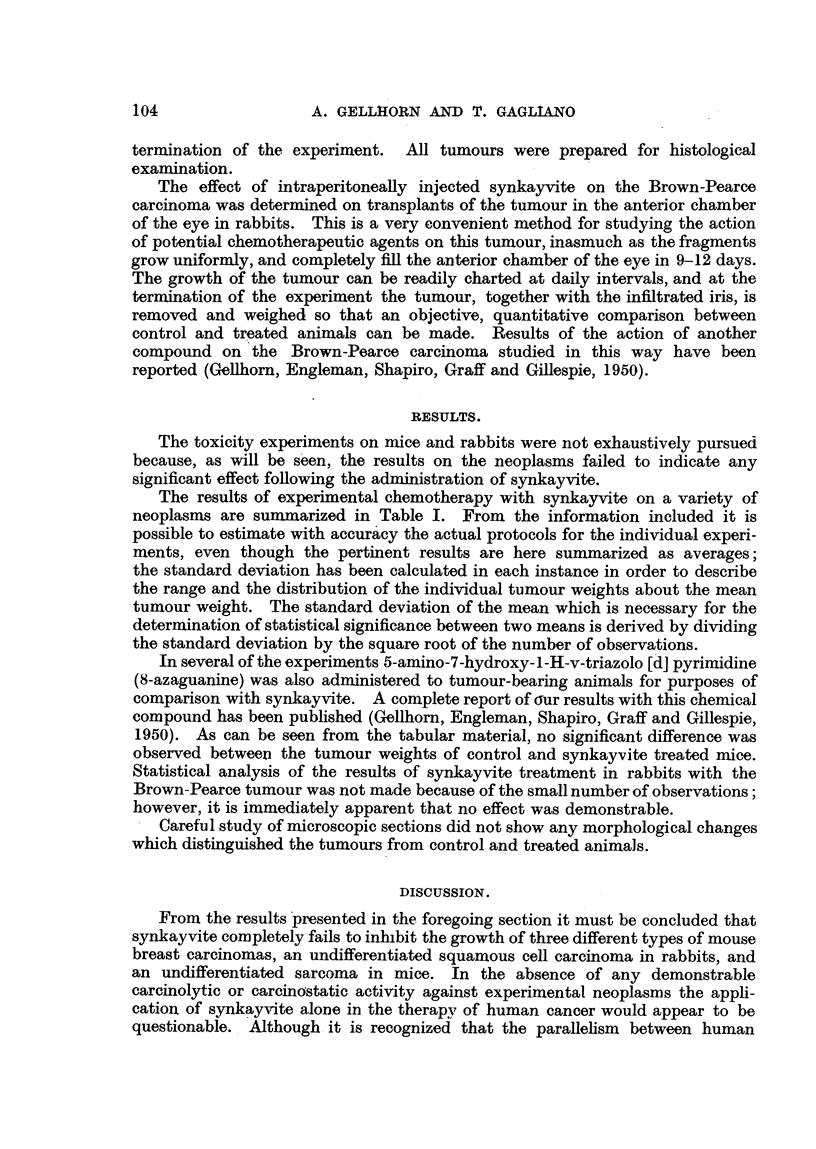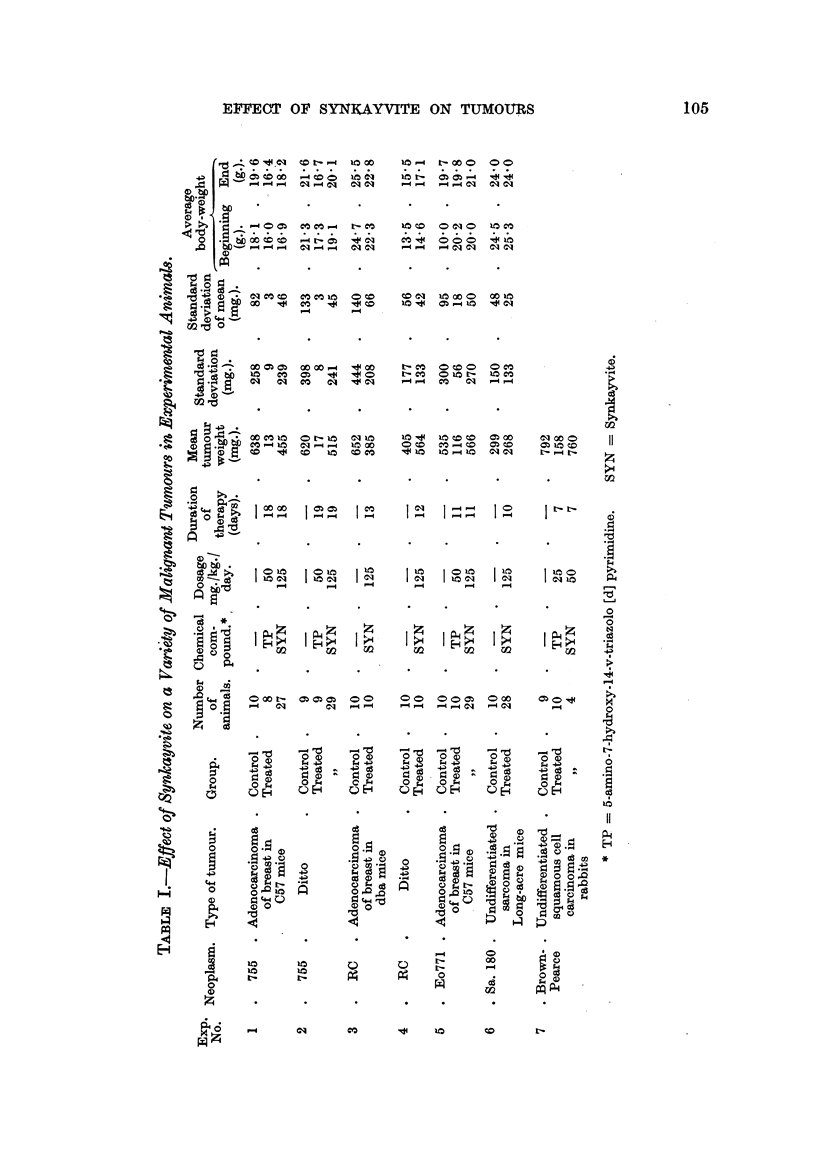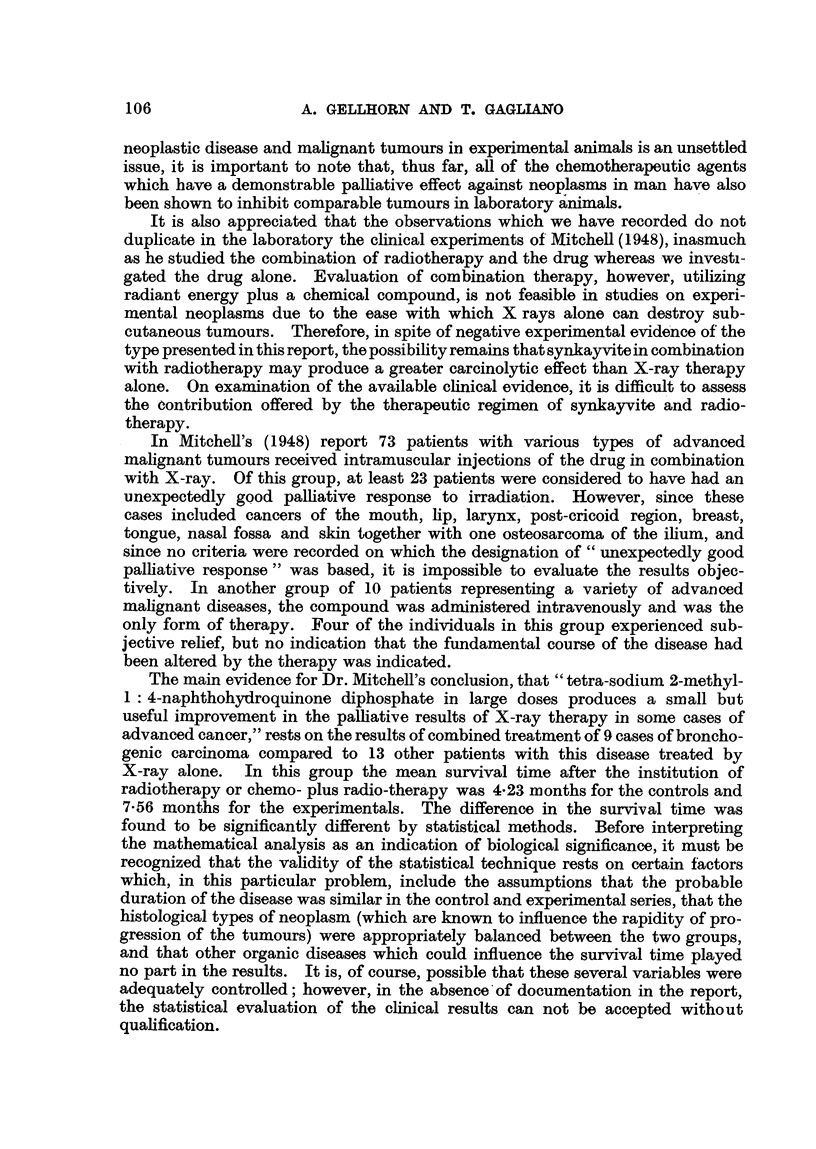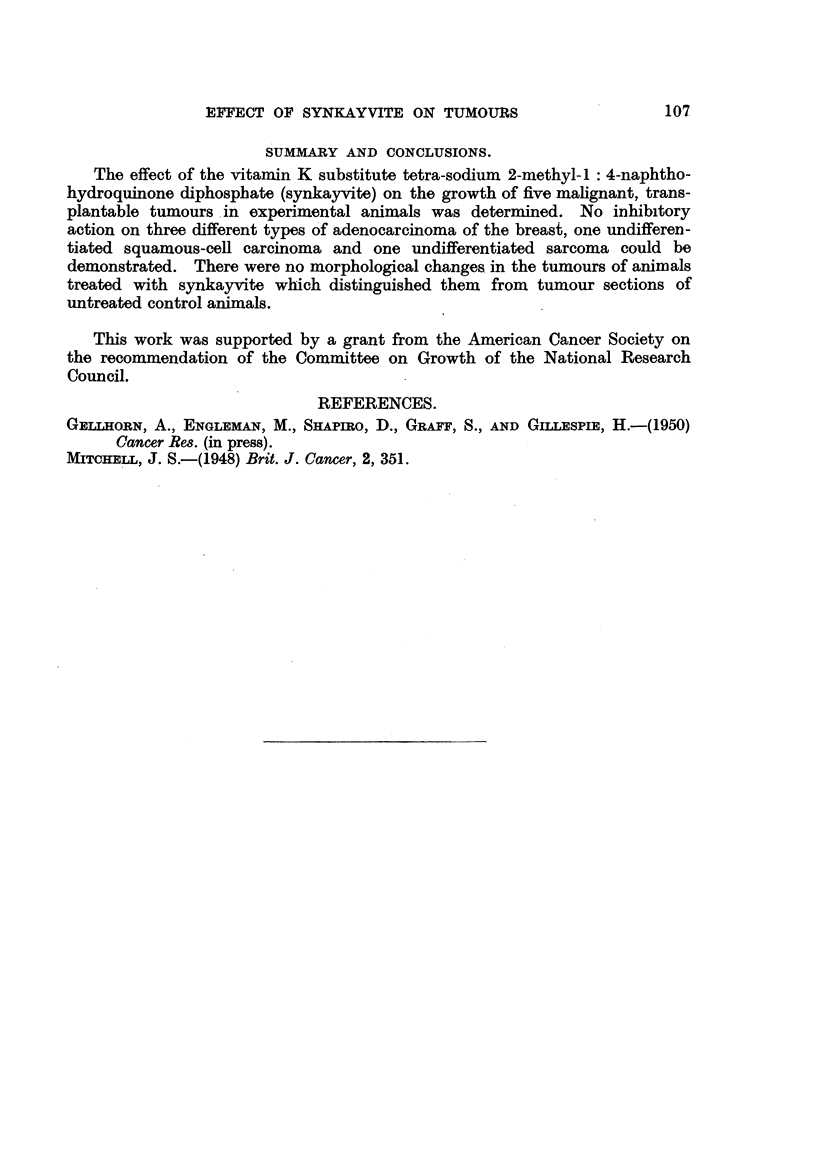# The Effect of Tetra-sodium 2-Methyl-1: 4-Naphthohydroquinone Diphosphate (Synkayvite) on a Variety of Malignant Tumours in Experimental Animals

**DOI:** 10.1038/bjc.1950.9

**Published:** 1950-03

**Authors:** A. Gellhorn, T. Gagliano


### THE EFFECT OF TETRA-SODIUM 2-METHYL-1 : 4-NAPHTHOHYDROQUINIONE DIPHOSPHATE (SYNKAYVITE) ON A VARIETY OF MALIGNANT TUMOURS IN EXPERIMENTAL ANIMALS.

A. GELLHORN AND T. GAGLIANO.

*From the Cancer Research Institute and the Department of Medicine, College of Physicians and Surgeons, Columbia University, New York.*

Received for publication January 30, 1950.

MITCHELL (1948) recently reported a clinical study on the use of parenterally administered tetra-sodium 2-methyl-1 : 4-naphthohydroquinone diphosphate (synkayvite) as an adjuvant to radiotherapy in the management of inoperable and far advanced malignant disease. In a small series he noted prolongation of the mean survival time of patients with inoperable carcinoma of the bronchus treated by the combination of chemical agent and irradiation when compared with that of patients who received X-ray therapy alone, which he found to be statistically significant.

The rationale for Mitchell's (1948) selection of this drug for clinical trial in combination with radiotherapy depended upon his observation that the chemical compound produced mitotic inhibition of chick fibroblasts in tissue culture.

Although there have been many laboratory studies on synkayvite as a vitamin K substitute, no investigations have been published on the effect of this compound on experimental neoplasms. On the basis of the claims made by Mitchell, it was considered of interest to determine whether synkayvite alone had any demonstrable effect on a variety of malignant tumours in experimental animals in order to provide a basis for clinical trials with the agent. This report will present the results of such experiments on four transplantable mouse tumours (three adenocarcinomas of the breast and an undifferentiated sarcoma), and the Brown-Pearce carcinoma in rabbits (undifferentiated squamous cell carcinoma).

### MATERIALS AND METHODS.

Preliminary chronic toxicity studies in mice were performed in the usual way to determine the maximal tolerated dose on which the animals could continue to maintain their body weight. Similar determinations were made in rabbits.

All of the mouse tumours were transplanted subcutaneously in the axillary region by the trocar method. The intraperitoneal administration of synkayvite was initiated 24 hours after transplantation, and continued daily for the duration of the experiment. Synkayvite was generously supplied by the Medical Division of Hoffman-La Roche, Inc., Nutley, N.J. The growth of the tumours in control and treated animals was followed by serial mensuration of the outside diameters, but the conclusions reached in this study rest on the comparison of the wet weight of the tumours in the control and treated series determined at the termination of the experiment. All tumours were prepared for histological examination.

The effect of intraperitoneally injected synkayvite on the Brown-Pearce carcinoma was determined on transplants of the tumour in the anterior chamber of the eye in rabbits. This is a very convenient method for studying the action of potential chemotherapeutic agents on this tumour, inasmuch as the fragments grow uniformly, and completely fill the anterior chamber of the eye in 9–12 days. The growth of the tumour can be readily charted at daily intervals, and at the termination of the experiment the tumour, together with the infiltrated iris, is removed and weighed so that an objective, quantitative comparison between control and treated animals can be made. Results of the action of another compound on the Brown-Pearce carcinoma studied in this way have been reported (Gellhorn, Engleman, Shapiro, Graff and Gillespie, 1950).

### RESULTS.

The toxicity experiments on mice and rabbits were not exhaustively pursued because, as will be seen, the results on the neoplasms failed to indicate any significant effect following the administration of synkayvite.

The results of experimental chemotherapy with synkayvite on a variety of neoplasms are summarized in Table I. From the information included it is possible to estimate with accuracy the actual protocols for the individual experiments, even though the pertinent results are here summarized as averages; the standard deviation has been calculated in each instance in order to describe the range and the distribution of the individual tumour weights about the mean tumour weight. The standard deviation of the mean which is necessary for the determination of statistical significance between two means is derived by dividing the standard deviation by the square root of the number of observations.

In several of the experiments 5-amino-7-hydroxy-1-H-v-triazolo [d] pyrimidine (8-azaguanine) was also administered to tumour-bearing animals for purposes of comparison with synkayvite. A complete report of our results with this chemical compound has been published (Gellhorn, Engleman, Shapiro, Graff and Gillespie, 1950). As can be seen from the tabular material, no significant difference was observed between the tumour weights of control and synkayvite treated mice. Statistical analysis of the results of synkayvite treatment in rabbits with the Brown-Pearce tumour was not made because of the small number of observations; however, it is immediately apparent that no effect was demonstrable.

Careful study of microscopic sections did not show any morphological changes which distinguished the tumours from control and treated animals.

### DISCUSSION.

From the results presented in the foregoing section it must be concluded that synkayvite completely fails to inhibit the growth of three different types of mouse breast carcinomas, an undifferentiated squamous cell carcinoma in rabbits, and an undifferentiated sarcoma in mice. In the absence of any demonstrable carcinolytic or carcinostatic activity against experimental neoplasms the application of synkayvite alone in the therapy of human cancer would appear to be questionable. Although it is recognized that the parallelism between human

TABLE I.—*Effect of Synkayvite on a Variety of Malignant Tumours in Experimental Animals.*

| Exp. No. | Neoplasm. | Type of tumour. | Group. | Number of animals. | Chemical compound.* | Dosage mg./kg./day. | Duration of therapy (days). | Mean tumour weight (mg.). | Standard deviation (mg.). | Standard deviation of mean (mg.). | Average body-weight Beginning (g.). | Average body-weight End (g.). |
|---|---|---|---|---|---|---|---|---|---|---|---|---|
| 1 | 755 | Adenocarcinoma of breast in C57 mice | Control | 10 | — | — | — | 638 | 258 | 82 | 18·1 | 19·6 |
| | | | Treated | 8 | TP | 50 | 18 | 13 | 9 | 3 | 16·0 | 16·4 |
| | | | | 27 | SYN | 125 | 18 | 455 | 239 | 46 | 16·9 | 18·2 |
| 2 | 755 | Ditto | Control | 9 | — | — | — | 620 | 398 | 133 | 21·3 | 21·6 |
| | | | Treated | 9 | TP | 50 | 19 | 17 | 8 | 3 | 17·3 | 16·7 |
| | | | ,, | 29 | SYN | 125 | 19 | 515 | 241 | 45 | 19·1 | 20·1 |
| 3 | RC | Adenocarcinoma of breast in dba mice | Control | 10 | — | — | — | 652 | 444 | 140 | 24·7 | 25·5 |
| | | | Treated | 10 | SYN | 125 | 13 | 385 | 208 | 66 | 22·3 | 22·8 |
| 4 | RC | Ditto | Control | 10 | — | — | — | 405 | 177 | 56 | 13·5 | 15·5 |
| | | | Treated | 10 | SYN | 125 | 12 | 564 | 133 | 42 | 14·6 | 17·1 |
| 5 | Eo771 | Adenocarcinoma of breast in C57 mice | Control | 10 | — | — | — | 535 | 300 | 95 | 10·0 | 19·7 |
| | | | Treated | 10 | TP | 50 | 11 | 116 | 56 | 18 | 20·2 | 19·8 |
| | | | ,, | 29 | SYN | 125 | 11 | 566 | 270 | 50 | 20·0 | 21·0 |
| 6 | Sa. 180 | Undifferentiated sarcoma in Long-acre mice | Control | 10 | — | — | — | 299 | 150 | 48 | 24·5 | 24·0 |
| | | | Treated | 28 | SYN | 125 | 10 | 268 | 133 | 25 | 25·3 | 24·0 |
| 7 | Brown-Pearce | Undifferentiated squamous cell carcinoma in rabbits | Control | 9 | — | — | — | 792 | | | | |
| | | | Treated | 10 | TP | 25 | 7 | 158 | | | | |
| | | | ,, | 4 | SYN | 50 | 7 | 760 | | | | |

* TP = 5-amino-7-hydroxy-14-v-triazolo [d] pyrimidine. SYN = Synkayvite.

neoplastic disease and malignant tumours in experimental animals is an unsettled issue, it is important to note that, thus far, all of the chemotherapeutic agents which have a demonstrable palliative effect against neoplasms in man have also been shown to inhibit comparable tumours in laboratory animals.

It is also appreciated that the observations which we have recorded do not duplicate in the laboratory the clinical experiments of Mitchell (1948), inasmuch as he studied the combination of radiotherapy and the drug whereas we investigated the drug alone. Evaluation of combination therapy, however, utilizing radiant energy plus a chemical compound, is not feasible in studies on experimental neoplasms due to the ease with which X rays alone can destroy subcutaneous tumours. Therefore, in spite of negative experimental evidence of the type presented in this report, the possibility remains that synkayvite in combination with radiotherapy may produce a greater carcinolytic effect than X-ray therapy alone. On examination of the available clinical evidence, it is difficult to assess the contribution offered by the therapeutic regimen of synkayvite and radiotherapy.

In Mitchell's (1948) report 73 patients with various types of advanced malignant tumours received intramuscular injections of the drug in combination with X-ray. Of this group, at least 23 patients were considered to have had an unexpectedly good palliative response to irradiation. However, since these cases included cancers of the mouth, lip, larynx, post-cricoid region, breast, tongue, nasal fossa and skin together with one osteosarcoma of the ilium, and since no criteria were recorded on which the designation of " unexpectedly good palliative response " was based, it is impossible to evaluate the results objectively. In another group of 10 patients representing a variety of advanced malignant diseases, the compound was administered intravenously and was the only form of therapy. Four of the individuals in this group experienced subjective relief, but no indication that the fundamental course of the disease had been altered by the therapy was indicated.

The main evidence for Dr. Mitchell's conclusion, that "tetra-sodium 2-methyl-1 : 4-naphthohydroquinone diphosphate in large doses produces a small but useful improvement in the palliative results of X-ray therapy in some cases of advanced cancer," rests on the results of combined treatment of 9 cases of bronchogenic carcinoma compared to 13 other patients with this disease treated by X-ray alone. In this group the mean survival time after the institution of radiotherapy or chemo- plus radio-therapy was 4·23 months for the controls and 7·56 months for the experimentals. The difference in the survival time was found to be significantly different by statistical methods. Before interpreting the mathematical analysis as an indication of biological significance, it must be recognized that the validity of the statistical technique rests on certain factors which, in this particular problem, include the assumptions that the probable duration of the disease was similar in the control and experimental series, that the histological types of neoplasm (which are known to influence the rapidity of progression of the tumours) were appropriately balanced between the two groups, and that other organic diseases which could influence the survival time played no part in the results. It is, of course, possible that these several variables were adequately controlled; however, in the absence of documentation in the report, the statistical evaluation of the clinical results can not be accepted without qualification.

### SUMMARY AND CONCLUSIONS.

The effect of the vitamin K substitute tetra-sodium 2-methyl-1 : 4-naphthohydroquinone diphosphate (synkayvite) on the growth of five malignant, transplantable tumours in experimental animals was determined. No inhibitory action on three different types of adenocarcinoma of the breast, one undifferentiated squamous-cell carcinoma and one undifferentiated sarcoma could be demonstrated. There were no morphological changes in the tumours of animals treated with synkayvite which distinguished them from tumour sections of untreated control animals.

This work was supported by a grant from the American Cancer Society on the recommendation of the Committee on Growth of the National Research Council.

### REFERENCES.

Gellhorn, A., Engleman, M., Shapiro, D., Graff, S., and Gillespie, H.—(1950) *Cancer Res.* (in press).

Mitchell, J. S.—(1948) *Brit. J. Cancer*, **2**, 351.